# Construction of an introgression line population for cultivated peanut (*Arachis hypogaea*) to facilitate breeding with wild relatives *Arachis batizocoi* and *Arachis stenosperma*

**DOI:** 10.3389/fpls.2026.1799510

**Published:** 2026-05-01

**Authors:** Emile C. Barnes, Soraya C.M. Leal-Bertioli, David J. Bertioli

**Affiliations:** 1 Institute of Plant Breeding, Genetics & Genomics, University of Georgia, Athens, GA, United States; 2 Department of Plant Pathology, University of Georgia, Athens, GA, United States; 3 Department of Crop & Soil Sciences, University of Georgia, Athens, GA, United States

**Keywords:** crop improvement, crop wild relatives, genetic resources, introgression breeding, introgression line population, peanut

## Abstract

Cultivated peanut is susceptible to several highly damaging pests, pathogens and abiotic stressors, largely due to its very narrow genetic base. However, a ploidy barrier exists between cultivated and highly resistant wild peanuts, which makes the process of interspecific hybridization relatively laborious and beyond the scope of many peanut breeding programs. Thus, the peanut breeding community would greatly benefit from a germplasm collection that captures the diversity represented by wild peanut species in a format that is compatible with cultivated peanut and easily introduced into breeding programs. To this end, we report the construction of a structured introgression line (IL) population bearing introgressions from the wild species *A. stenosperma* and *A. batizocoi*. The IL population, which has 32 lines in total, has been constructed to maximize genome coverage across the cultivated peanut allotetraploid genome with complementary introgressed segments of a mean size of 34.7 Mbp. Nine of 10 chromosomes in the A subgenome and six of 10 in the B subgenome have at least one introgression. 60.1% of polymorphic markers from *A. stenosperma* and 34.8% from *A. batizocoi* are covered by introgressions. The phenotypic diversity of the population is demonstrated through several traits, including disease resistance and plant height. This IL population will be released for public use to the broader peanut research and breeding community. The open-ended nature of this resource will allow researchers and breeders to exploit the genetic value of these two wild peanut relatives for a number of traits without themselves needing to take on the task of interspecific hybridization.

## 1 Introduction

Cultivated peanut (*Arachis hypogaea* L.) is a major food, feed, and oilseed crop grown in tropical and subtropical regions around the world. It is the second most widely-grown legume globally, second only to soybean; in 2019, an estimated 53.9 million tons were harvested from 32.7 million hectares for an average global yield of 1.65 t/ha (Food and Agriculture Organization of the United Nations, 2023). Despite its widespread importance as both a cash crop and essential source of dietary protein and calories in varying parts of the world, peanut production remains severely hampered by several major pests, pathogens, and abiotic factors that can greatly reduce yield.

Efforts to breed for resistance or tolerance to these varying limiters of yield have been undermined by the severe lack of diversity in the primary gene pool of cultivated peanut. This lack of diversity reflects the origin of cultivated peanut. The native range of the *Arachis* genus extends across a wide area of South America, from northeastern Brazil south into the Argentine Espinal and westward to the Eastern slopes of the Andes mountains. It is near the conjunction of southeastern Bolivia, northeastern Paraguay, and northern Argentina that domesticated peanut is currently considered to have arisen from the hybridization of the diploid species *A. duranensis* and *A. ipäensis*, contributing the A and B subgenomes, respectively, of a sterile hybrid. Following spontaneous genome duplication, the wild ‘AABB’ allotetraploid A*. monticola* was formed, from which *A. hypogaea* was domesticated; they are the only two tetraploid members of the genus. The bottleneck effect resulting from this origin is twofold in that its formation was a unique event, limited to the genomic contributions of only two particular accessions of the wild progenitor species, and that tetraploidization prevented gene flow between the nascent tetraploids and the other diploid members of the genus (Bertioli et al., 2011, Bertioli et al., 2019, Bertioli et al., 2020; Rami et al., 2014; De Blas et al., 2025).

Consequently, breeders seeking to improve the resilience of peanut, particularly disease resistance, have found the greatest success in the introduction of alleles from wild members of the genus *Arachis*. However, this process requires breeders to invest a considerable amount of time in overcoming the ploidy barrier between cultivated peanut and its wild relatives, which is accomplished through either the so-called tetraploid or hexaploid routes. The tetraploid route involves the formation of an ‘AABB’ wild species-derived induced allotetraploid sexually compatible with cultivated peanut by crossing an ‘A’ genome and ‘B’ genome wild species, treating the sterile hybrid with colchicine, and multiple rounds of backcrossing to cultivated peanut. The hexaploid route generates a cytogenetically unstable hexaploid by crossing the wild diploid to cultivated peanut and treating the resultant sterile triploid with colchicine, followed by several generations of selfing until the tetraploid state is recovered (Bertioli et al., 2011). Either process takes a minimum of 1.5 years, but often more, and is generally performed for the targeted introduction of a specific trait of interest, with the remaining wild genomic contributions purged through continual backcrossing cycles. The potential value of these discarded alleles is often not considered.

The value of an introgression line population, particularly for peanut, is that the upfront effort in wild-derived breeding material sexually compatible with cultivated peanut and the initial cycles of backcrossing have already been done with the genetic contributions of the wild species discretized in a collection of lines easily crossed to the cultivated species. While each line contains little wild genetics, the entire population collectively captures most of the wild genome. Thus, breeders can assay the population for any trait of interest that they suspect may be present in the wild donor species, and upon identifying lines within the population with the desired phenotype, immediately begin crosses with elite material to introduce the beneficial allele into their breeding program.

To date, one such population has been created for peanut, using the wild progenitors of cultivated peanut, *A. duranensis* and *A. ipäensis*, as the donor species, with essentially complete representation of both wild genomes in the genetic background of the Spanish-type cultivar ‘Fleur 11’ (Fonceka et al., 2012). This landmark achievement created a resource that has proved useful for several studies involving genetic mapping and basic genetics research in peanut, leading to the identification of QTLs for pod and seed size (Alyr et al., 2020); pod number, pod weight, total biomass and other yield component traits (Tossim et al., 2020); seed oil composition (Gimode et al., 2020); leaf spot resistance (Gimode et al., 2023); and chlorophyll content, measured as a corollary to nitrogen fixation (Gimode et al., 2023). Its use has also been applied to peanut breeding in Senegal, where six cultivars developed using these introgression lines have been released with yields up to three times higher than traditional local lines due to higher genetic yield potential and adaptation to high rainfall conditions (Desmae et al., 2016; Conde et al., 2024; Sy, 2018). However, because of the recency of tetraploidization from these two species and the severe founder effect discussed above, *A. duranensis* and *A. ipäensis* retain high degrees of sequence similarity to the corresponding subgenomes of *A. hypogaea* and are suboptimal donors in terms of increasing the diversity of the cultivated peanut gene pool.

In the present study, we report the production of a novel introgression line (IL) population with the wild donor species *A. stenosperma* and *A. batizocoi*, which demonstrate several traits useful for peanut breeding, most notably resistance to many common diseases and pathogens (Stalker, 2017). In addition to the remarkable utility of an IL population as a genetic tool as demonstrated by the extensive use of the peanut scientific community’s one such resource, we believe that the more distant relationship of the two wild species represented in these introgression lines will provide further value in broadening the genetic basis of this global staple crop.

## 2 Materials and methods

### 2.1 Population development

Wild peanut accessions *A. batizocoi* PI298639 (collection number K9484) and *A. stenosperma* PI666100 (collection number V10309) were crossed to form a sterile diploid hybrid, which was treated with colchicine to form a fertile allotetraploid (‘BatSten1’) sexually compatible with *A. hypogaea* (Bertioli et al., 2021b). Three rounds of backcrossing to *A. hypogaea* were performed in greenhouse conditions (Ballén-Taborda et al., 2022). Breeding line ‘5-646-10’, derived from the cross ‘Florida-07’ × ‘Tifguard’, was the primary recurrent parent, although breeding lines ‘13-1014’ (derived from [C1805-617-1 (‘Florida-07’ x ‘Tifguard’) x ‘Georgia-06G’]) and ‘13-2113’ (derived from [C1805-2-9-16 (‘Florida-07’ x ‘Tifguard’) x ‘TifGP-2’]) were used in the BC_2_ and BC_3_ generations for some lines. These breeding lines were chosen as parents for their high oleic/linoleic acid ratios and, in the case of ‘5-646-10’, for its relatively high yield for a breeding line. Full pedigrees for each line are given in **Supplementary Table 1** “RBS-IL_genotyping.xlsx”.

KASP markers were developed based on SNP markers specific to *A. stenosperma* or polymorphic between ‘BatSten1’ and *A. hypogaea* from the ‘Axiom_Arachis’ 48K high-density SNP array v.02 (Clevenger et al., 2018; Korani et al., 2019). Because the parent population was originally developed to introduce root-knot nematode resistance into cultivated germplasm, 16 markers on chromosomes A02 and A09 associated with RKN resistance were used to perform foreground selection in the first three backcross cycles (Ballén-Taborda et al., 2022).

In 2021, 74 lines from the BC_3_F_2_, BC_2_F_2_, and BC_1_F_2_ generations were selected to maximize introgression coverage across the cultivated peanut genome and four randomly selected seeds per line were planted at the Southeast Georgia Research and Education Center in Midville, GA. Plants from each line were harvested without selection and bulk-harvested together to maintain the heterogeneity of each line. In 2022, eight randomly selected seeds per line were again planted in Midville, GA and harvested in bulk. In 2023, one mini-row (12’ plot of bifurcate rows planted at six seeds per foot) and four randomly selected single seeds were planted in Midville, GA, with single plants genotyped and harvested. Also in 2023, five new lines from the same backcross parent population that were concurrently advanced and selected for nematode resistance and agronomic performance were introduced into the IL population. From the seeds from these plants and their genotyping data, a selection of 32 lines was made to provide maximal wild genome introgression coverage with minimal “off-target” introgressions, preferring a smaller population size for greater ease of use. The final population of 32 lines was selected and planted in triplicate mini-rows in Midville, GA in 2024. Of the final population, one line is of BC_4_F_6_ seed (‘RBS-IL_A02-3’), five are of BC_2_F_6_ seed (‘RBS-IL_A01-2’, ‘RBS-IL_A03-1’, ‘RBS-IL_A04-3’, ‘RBS-IL_B03-1’, and ‘RBS-IL_B09-1’), and the remaining lines are all of BC_3_F_6_ seed.

### 2.2 Genotyping

For each line, unexpanded young leaves were collected and lyophilized. Approximately 20 mg of lyophilized tissue was processed using the Qiagen^®^ DNeasy^®^ Plant Mini Kit (catalog number 69104). Samples were genotyped with the ‘Axiom_Arachis’ 48K high-density SNP array v.02 (Clevenger et al., 2018; Korani et al., 2019) by Thermo Fisher. Polymorphic SNPs were identified as introgressions based on parental genotyping with the criteria of ‘*A. stenosperma* ≠ *A. hypogaea* OR *A. batizocoi*’ and ‘*A. batizocoi* ≠ *A. hypogaea* OR *A. stenosperma*’. Genetic map distance was determined using JoinMap (Stam, 1993).

### 2.3 Phenotypic evaluation

In 2024, mini-rows were evaluated for incidence of tomato spotted wilt virus (TSWV), early and late leaf spot (ELS/LLS, scored together simply as ‘leaf spot’) and flower color. TSWV was scored visually on a 0–5 scale, where 0 = no symptoms; 1 = minimal symptoms without stunting; 2 = moderate symptoms with mild stunting; 3 = moderate symptoms with noticeable stunting; 4 = high symptoms with high stunting; and 5 = severe symptoms (including plant death) with severe stunting (Baldessari, 2008). These scores were used to calculate average severity per genotype and incidence, scored as the percentage of plants scored 2 or higher. Leaf spot was scored using the 1–10 Florida scale, where 1 = no leaf spot; 2 = very few lesions, none on upper canopy; 3 = few lesions, very few on upper canopy; 4 = some lesions, more on upper canopy, 5% defoliation; 5 = lesions noticeable even on upper canopy, 20% defoliation; 6 = lesions numerous and evident on upper canopy, 50% defoliation; 7 = lesions numerous on upper canopy, 75% defoliation; 8 = upper canopy covered with lesions, 90% defoliation; 9 = very few leaves remaining, covered in lesions, 98% defoliation; 10 = plants are completely defoliated and/or killed by leaf spot (Chiteka et al., 1988). Evaluations were made at 113, 133 and 148 DAP and used to calculate the area under the disease progression curve (AUDPC) (Jeger and Viljanen-Rollinson, 2001). Flower color was evaluated visually at 43 DAP scored as either yellow (1) or orange (0).

Harvested mini-rows were cleaned of debris and weighed using a kilogram scale. After harvest, pod and seed data were collected for each line in the population. Hundred-seed weight was measured as the total weight in grams of 100 randomly selected seeds. Ten pods per line were randomly selected and pod constriction between the two pod halves was evaluated on a 1–4 scale, where 1 = very deep constriction; 2 = deep constriction; 3 = moderate constriction; 4 = no constriction (modified from Ballén-Taborda et al., 2024). Unmanned aerial vehicle (drone) images were taken roughly every 2 weeks using a DJI Mavic 3 Enterprise RTK flown at a height of 30 m. Orthomosaic images were generated using Pix4Dmapper and analyzed using FIELDimageR software (Matias et al., 2020).

The detached ELS leaf bioassay was conducted as described in Leal-Bertioli et al., 2009 and Gonzales et al., 2023. Briefly, nine fully expanded leaves were collected from each plant. Leaves with their petioles wrapped in wet cotton were mounted in 100 × 15 mm Petri dishes (VWR International, LLC, Radnor, PA) containing a wet cotton pad covered by a 9.0-cm diameter P5 Fisherbrand filter paper (Fisher Scientific Co., L.L.C., Pittsburgh, PA) and a 25 × 75 × 1 mm microscope slide (Fisher Scientific Co., L.L.C., Pittsburgh, PA). ELS strain ‘E13B’, a single-spore isolate initially collected from Tift County, Georgia, was used for inoculation. To inoculate, sporulating culture plates were washed with a 0.005% solution of Tween-20 and gently scraped with an L-shaped cell spreader to make a spore suspension. The concentration of spores was measured by hemocytometer and diluted as necessary to 25,000 spores/mL. Inoculation was performed by brushing the upper leaf surfaces with a small paintbrush dipped in the spore suspension. After being allowed to air-dry until completely dry, Petri dish chambers were closed and kept in darkness for 48 hours before being moved to growth chambers. Leaves were kept in growth chambers for the remainder of the bioassay for the observation of disease progression. Growth chamber conditions were 12h:12h light/dark cycle, 25-27 °C temperature, and 40-60% relative humidity. Leaves were initially monitored daily to record the days until the first visible lesion(s) (incubation period) for each genotype. After the appearance of lesions, leaves were checked every other day to count the number of total lesions and the number of sporulating lesions. The endpoint of the bioassay was determined as the point at which the most severely affected leaves were beginning to decay or show secondary infection. Lesion number AUDPC values were calculated as above. The identity of the pathogen as ELS rather than LLS was confirmed by examination of conidiophore morphology at the time of inoculation and the pattern of lesion development (i.e., first appearance on adaxial leaf surface, characteristic “halo” of chlorotic tissue surrounding lesions, 6–12 day incubation period rather than 15–21 days). At the conclusion of the experiment, leaf images were collected using a flatbed scanner and leaf area was measured using APS Assess 2.0 (Lamari and American Phytopathological Society, 2008).

### 2.4 QTL identification

Genotypic and phenotypic data were imported into R Statistical Software version 4.3.2 (R Core Team, 2021). QTL analysis was performed using the r/qtl2 package (Broman et al., 2019). To account for low marker density, pseudomarkers were inserted in the genetic map at 1 cM intervals and genotypes predicted using the calc_genoprob() function. Interval mapping approximation by Haley-Knott regression was performed using the scan1() function. Putative QTLs were identified using find_peaks() with standard parameters (LOD threshold of 3) and 95% Bayes credible intervals were identified with the bayes_int() function. The fit1() function was used to estimate QTL effects and PVE values.

### 2.5 Data analysis, visualization and statistical methods

**Figure 1:**
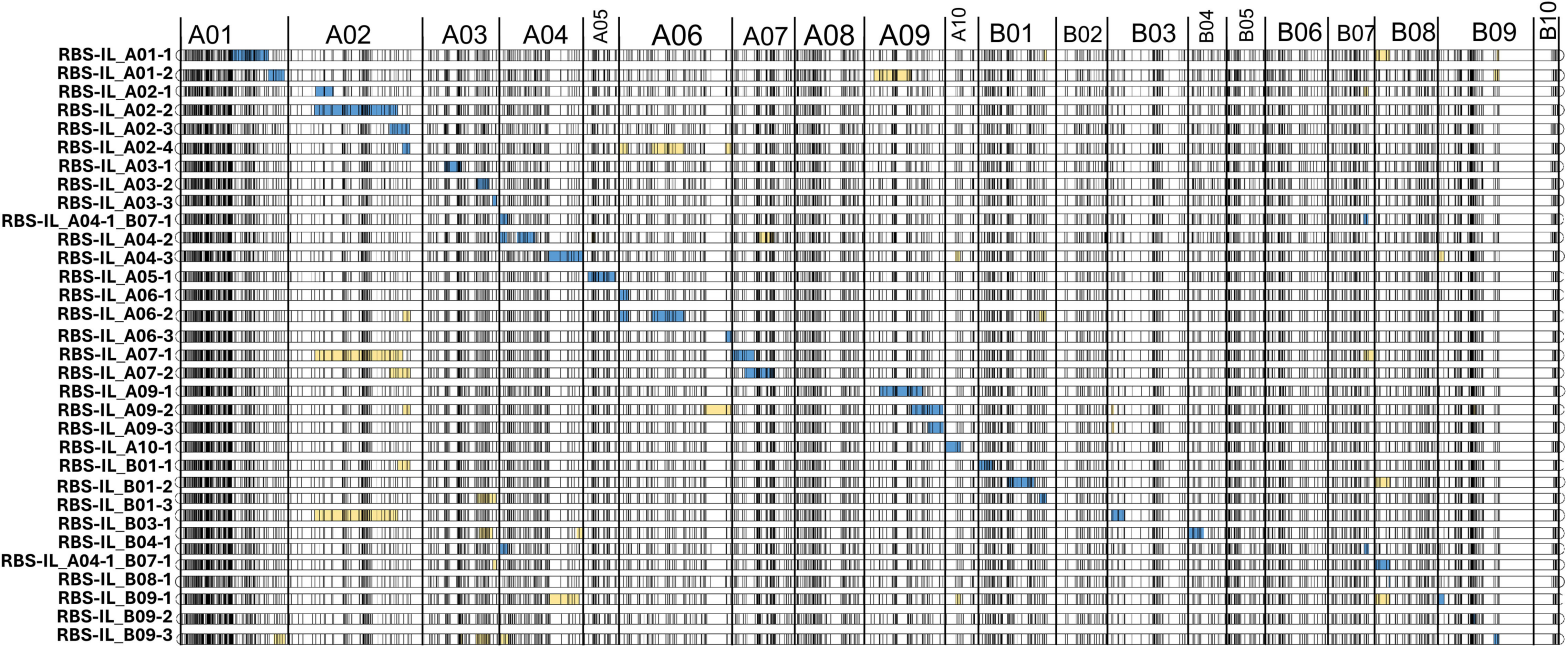
Genome map of *Arachis hypogaea* × ‘BatSten1’ introgression line population. SNP markers are arranged horizontally by chromosome in recombination distance (cM), going left-to-right. Each row represents an introgression line where blue bars show the introgressed region on which inclusion in the population was based. Yellow bars indicate off-target introgressions. Figure formatted using the LinkageMapView package for R (Ouellette et al., 2018).

**Figure 2:**
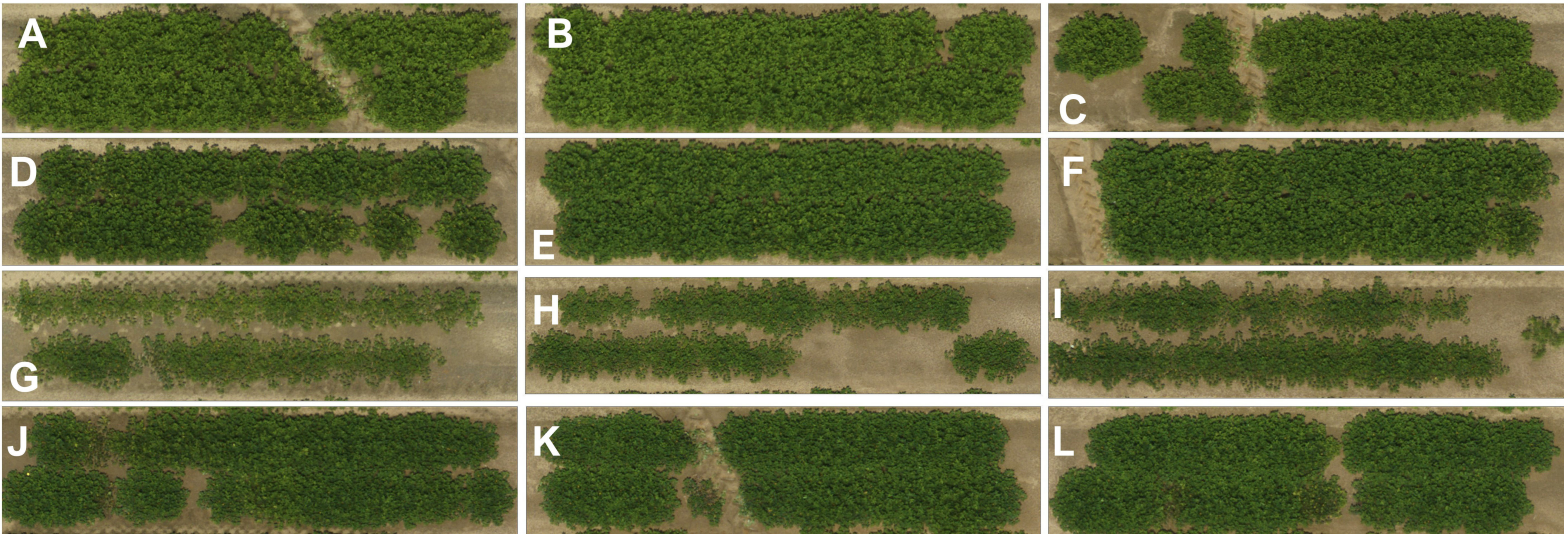
Aerial imagery taken 81 DAP compares growth habit of introgression lines with controls. ‘RBS-IL_B01-2’ **(D–F)** shows similar growth habit and mean plant height to cultivar ‘Bailey II’ **(A–C)** of the Virginia botanical type. Introgression line ‘RBS-IL_A07-1’ **(G–I)** shows a greatly reduced stature compared to the recurrent parent, ‘5-646-10’ **(J–L)**.

All analyses were performed using R Statistical Software version 4.3.2 (R Core Team, 2021). Histograms and bar graphs were generated using ggplot2 (Wickham, 2016). Chromosome map in **Figure 1** was generated using R package LinkageMapView (Ouellette et al., 2018). Unmanned aerial vehicle (drone) images taken using a DJI Mavic 3 Enterprise RTK flown at a height of 30 m are used in **Figure 2**. Differences of means were computed by Student’s *t*-test unless otherwise indicated.

## 3 Results

### 3.1 Introduction of *A. stenosperma* and *A. batizocoi* genetics in peanut introgression lines

Alleles from the wild donor species were successfully identified in 72 of 76 lines planted in 2023, spanning fifteen of twenty chromosomes. Based on genotyping results, a subset of 32 lines representing the final IL population were chosen from single plants to maximize genomic coverage with homozygous wild introgressions. Single plants were necessarily selected based on survival in the field and adequate seed yield to plant a replicated field trial in 2024; thus, these ILs show acceptable vigor for small-scale experimental and breeding purposes, although strong vigor was not a selection criterion.

Across all ILs, the average genomic composition is 97.2% cultivated and 2.8% wild, with an average introgression size of 34.7 Mbp and a mean 2.4 introgressions per line (median: 2). In total, 12 lines have one introgression; seven lines have two introgressions; six lines have three introgressions; four lines have four introgressions; and three lines have five introgressions. Introgressions cover 60.1% of markers in the A subgenome and 34.6% of markers in the B subgenome, for an estimated average of 49.8% of the full cultivated genome covered by at least one introgression. One line, ‘RBS-IL_A04-1_B07-1’, was selected to cover portions of both chromosomes A04 and B07 because other lines covering these chromosomes had higher amounts of off-target introgression (defined as introgressions other than the one for which inclusion in the population was based on, e.g., a segment on chromosome A09 in line ‘RBS-IL_A01-2’). Tabular and graphic representations of coverage for each chromosome are shown in **Table 1** and **Figure 1**. Full genotyping information is presented in **Supplementary Table 1** “RBS-IL_genotyping.xlsx”.

**Table 1:** Average introgression size and coverage per chromosome across all lines representing that chromosome in *A. hypogaea*.

Chromosome	Avg. introgression size	Coverage	Chromosome	Avg. introgression size	Coverage
A01	55.38 Mbp	33%	B01	45.15 Mbp	77%
A02	30.74 Mbp	89%	B02	0 Mbp	0%
A03	30.01 Mbp	82%	B03	12.12 Mbp	24%
A04	21.07 Mbp	66%	B04	70.61 Mbp	41%
A05	90.60 Mbp	86%	B05	0 Mbp	0%
A06	28.63 Mbp	58%	B06	0 Mbp	0%
A07	30.41 Mbp	82%	B07	5.15 Mbp	13%
A08	0 Mbp	0%	B08	15.82 Mbp	26%
A09	10.84 Mbp	91%	B09	25.61 Mbp	50%
A10	48.28 Mbp	38%	B10	0 Mbp	0%

Coverage is estimated as the percentage of polymorphic SNPs on a chromosome at which the wild allele is present in the IL targeting that region.

### 3.2 Population genetic diversity

**Figure 3:**
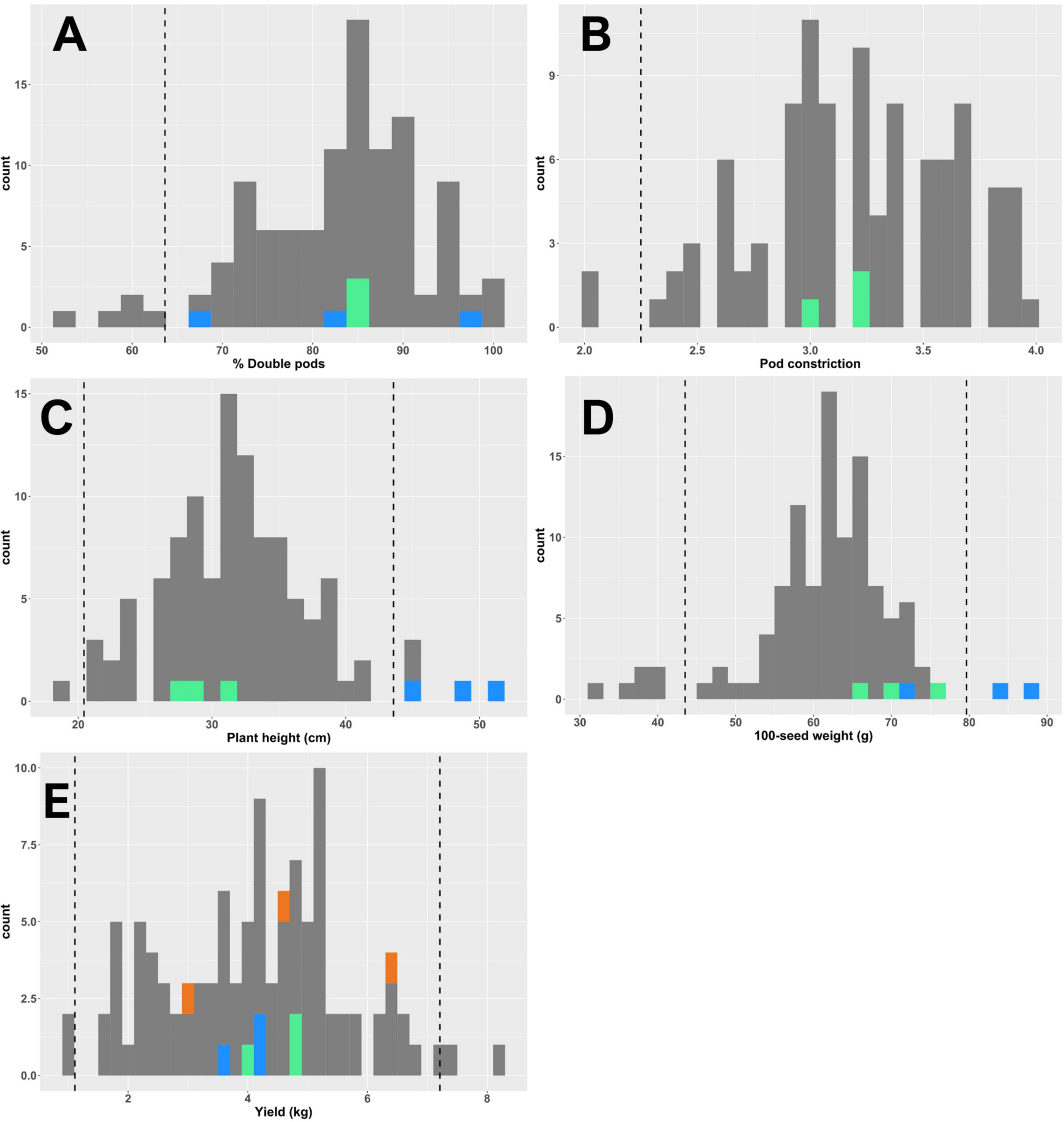
Histograms displaying distributions of phenotypes for percentage of double pods **(A)**, pod constriction **(B)**, plant height **(C)**, 100-seed weight **(D)**, and yield **(E)**. In each panel, values for each of three replications are depicted. Gray values represent introgression lines; green values represent the recurrent parent ‘5-646-10’; blue values represent a Virginia-type cultivar, ‘Bailey II’, notable for its tall stature and large seeds typical of its botanical type; and orange values represent ‘Georgia-06G’, the most commonly grown cultivar in the Southeast. Dashed lines indicate the thresholds beyond which values are statistically significant at *p* < 0.05, based on a two-tailed Student’s *t*-test.

**Figure 4:**
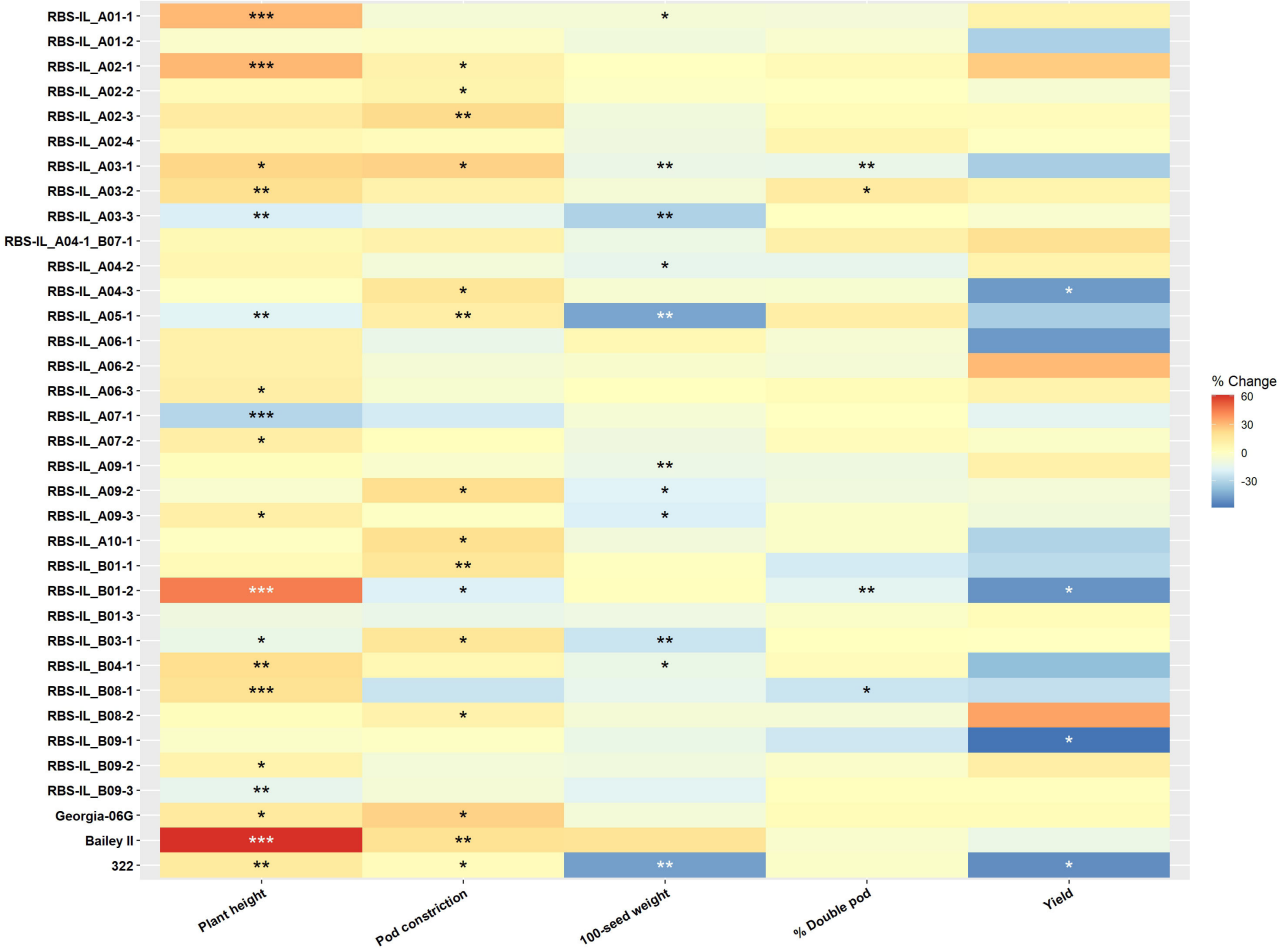
Heatmap of phenotypic variance among introgression lines compared to the recurrent parent ‘5-646-10’, shown as a percentage difference from the recurrent parent phenotypic value. Statistical significance of the difference is indicated by *p < 0.05; **p < 0.01; ***p < 0.001 according to a Student’s t-test. Control lines ‘Georgia-06G’, ‘Bailey II’ and ‘IAC 322’ are shown for comparison. Full phenotyping data is available in **Supplementary Materials**.

For all end-season pod and seed traits measured—including hundred-seed weight, pod constriction, percentage of double pods, and yield—as well as mid-season plant height, the introgression lines exhibited a normal distribution of phenotypes approximately centered around the phenotype of the main recurrent parent, line ‘5-646-10’. Histograms depicting population phenotypic values for these traits are shown in **Figure 3**. The distribution of phenotypic values and statistical separation among some lines from the recurrent parent demonstrates the successful introduction and fixation of diversity from the genetic donor, ‘BatSten1’. Full phenotypic information is presented in **Supplementary Table 2** “RBS-IL_phenotyping.xlsx”. A heatmap of phenotypic departures from the recurrent parent is presented in **Figure 4**.

Plant height shows a notable degree of variation within the population. The introgression line ‘RBS-IL_B01-2’ had a mean central stem height of 43.1 ± 1.14 cm, not statistically significantly different from the cultivar ‘Bailey II’ (mean height 47.8 ± 1.00 cm), which belongs to the Virginia botanical type, despite no genetic relation. Cultivars of this botanical type are notably much taller and larger-seeded plants than the Runner-type plants such as ‘5-646-10’ (mean height 29.7 ± 0.65 cm). Four other lines also show highly statistically significant increases in mean plant height (*p* < 0.01). In contrast, four lines show comparably strong reductions in plant height (*p* < 0.01), with the lowest mean plant height of 21.0 ± 0.57 cm in ‘RBS-IL_A07-1’. The difference in growth habit between these tallest and shortest introgression lines can be appreciated visually in **Figure 2**.

### 3.3 Plant utility in genetic mapping

**Figure 5:**
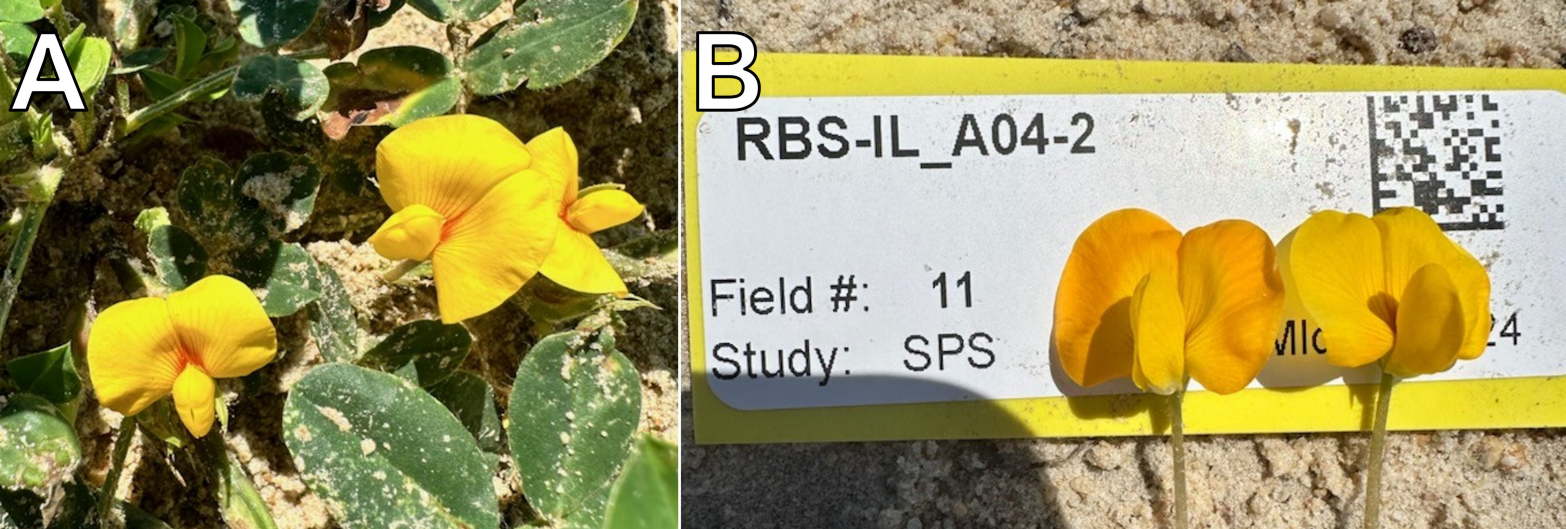
Two lines exhibit yellow flowers: ‘RBS-IL_A05-1’ **(A)**, in which yellow flowers are homozygous, and ‘RBS-IL_A04-2’ **(B)**, which segregates 1-to-1 for yellow and orange flowers. The lines overlap in a region of chromosome A05 previously implicated in control of flower color (Fonceka et al., 2012).

We have used flower color to demonstrate the functionality of this population in quickly and easily mapping qualitative Mendelian traits. In the genus *Arachis*, wild species have both yellow and orange flowers while the cultivated species has exclusively orange flowers. Both wild donors to this population, *A. stenosperma* and *A. batizocoi*, have yellow flowers. In our population, all ILs show orange flowers except for two, ‘RBS-IL_A05-1’ and ‘RBS-IL_A04-2’. The former has all yellow flowers while the latter has orange and yellow flowers in an approximate 1-to-1 ratio (**Figure 5**). The two lines overlap in a 1.8 Mbp segment of chromosome A05, between 9.1 and 10.9 Mbp, for which ‘RBS-IL_A05-1’ is homozygous and ‘RBS-IL_A04-2’ is heterozygous. Thus, we deduce the genetic control for flower color to lie within this interval.

For the trait of pod constriction, we sought to compare our results to those previously found in a genetic mapping and QTL validation study performed in similar lines derived from the same larger population of backcross lines with ‘BatSten1’ genetics (Ballén-Taborda et al., 2024). In the previous study, a 3.7 Mbp wild introgression at the top of chromosome B08 was found to explain 47.3% of phenotypic variation. The introgression line intended to target this chromosomal region, ‘RBS-IL_B08-1’, had the lowest average pod constriction score among all lines (2.37 ± 0.20), indicating the most severe constriction on a 1–4 scale, consistent with previous findings. However, this did not differ significantly from the recurrent parent (3.13 ± 0.07; p = 0.073), possibly due to a relatively high level of variance within the sample. One line, ‘RBS-IL_B01-2’, was significantly more constricted than the RP with a mean score of 2.53 ± 0.07 (p = 0.035). This line harbors its target introgression on chromosome B01 but also has an ‘off-target’ introgression at the top of chromosome B08, covering this 3.7 Mbp QTL identified by Ballén-Taborda et al. (2024).

To measure quantitative trait loci more robustly, QTL mapping was performed for pod constriction as well as plant height in centimeters, yield per plot in kilograms, 100-seed weight in grams, and percentage of double pods. Two QTL were identified for each trait except hundred-seed weight, for which one QTL was found. These results are summarized in **Table 2**.

**Table 2:** Quantitative trait loci (QTL) identified in 2024 phenotyping data of IL population plant and pod/seed traits.

Trait	QTL	Chr.	Effect size	Mean PVE	LOD score	Position, cM (Mbp)	Confidence interval (cM)
Plant height (cm)	PH_A07_1	A07	-10.97	23.4%	3.10	8.15 (4.05)	0.00 – 167.50
	PH_B01_1	B01	12.05	23.90%	4.37	137.302 (131.91)	45.44 – 171.49
Yield (kg)	Yield_A04_1	A04	-1.01	19.9%	3.54	180.68 (134.73)	37.30 – 245.50
	Yield_B09_1	B09	-2.37	18.4%	3.26	8.31 (2.10)	0.00 – 115.31
Pod constriction (1–4 scale)	PC_A03_1	A03	-0.73	23.4%	4.26	144.08 (124.26)	35.44 – 242.26
	PC_B08_1	B08	-0.52	19.7%	3.55	31.80 (27.61)	0.00 – 42.60
100-seed weight (g)	SW_A05_1	A05	-24.52	46.9%	27.28	70.86 (102.00)	0.00 – 100.69

Chr., chromosome; PVE, percent of variation explained. Effect sizes are given in the same units that the trait value is reported in, listed under each trait name.

A QTL for pod constriction overlapping that identified by Ballén-Taborda et al. (2024) was detected (*PC_B08_1*), as well as a second, *PC_A03_1*, which may be a small-effect QTL inflated by apparent genetic linkage to *PC_B08_1*. The two QTLs identified for plant height, *PH_A07_1* and *PH_B01_1*, had similar magnitudes of effect size (-10.97 and +12.05 cm, respectively) and percentage of variation explained (PVE) values (23.4% and 23.9%). These QTLs localize to the introgressions covered by the ILs with the greatest phenotypic difference in plant height from the recurrent parent, ‘RBS-IL_A07-1’ and ‘RBS-IL_B01-2’. Both yield QTL were associated with negative penalties accountable for 18.4-19.9% phenotypic variation, although the magnitude of effect was less for *Yield_A04_1* (-1.01 kg/plot) than *Yield_B09_1* (-2.37 kg/plot). The most significant QTL was seen for hundred-seed weight, *SW_A05_1*, in a region that was previously implicated in seed weight in peanut (Gangurde et al., 2020). We found an average reduction of 24.52 g/hundred seeds, corresponding to 46.9% of PVE.

### 3.4 Disease resistance

As previously discussed, the donor species *A. stenosperma* and *A. batizocoi* were selected in part because of their demonstrated resistance to several major pathogens of cultivated peanut (Seijo et al., 2007). In this population, *A. stenosperma* introduces strong and novel resistance genes to root-knot nematode (*Meloidogyne arenaria*) as reported in (Ballén-Taborda et al., 2019, Ballén-Taborda et al., 2022; Barnes et al., 2025). Two lines, ‘RBS-IL_A01-2’ and ‘RBS-IL_A06-1’, may also harbor a small peanut rust (*Puccinia arachidis*) resistance locus previously identified at the top of chromosome B02 (unpublished results). Presently, this signal at one SNP cannot be distinguished from genotyping error and warrants testing through either field or *in vitro* study. Identification of more SNPs in this genomic region capable of differentiating introgressions from *A. batizocoi* would additionally help efforts to investigate this potential source of rust resistance.

In our 2024 field trial, replicated mini-rows (*n* = 3) were evaluated for TSWV and leaf spot four times and three times throughout the growing season, respectively. Two lines, ‘RBS-IL_B08-1’ and ‘RBS-IL_B08-2’, showed moderate reductions in end-of-season TSWV severity, -35% and -25% respectively (mean score 2.17 ± 0.17, 2.50 ± 0.29; *p* = 0.069, 0.190) compared to recurrent parent ‘5-646-10’ (mean score 3.33 ± 0.44). The moderate resistance of these lines is more notable compared to the leading cultivar ‘Georgia-06G’ (mean score 3.50 ± 0.29; *p* = 0.015 and 0.074, respectively).

Contrarily, we found no significant results for leaf spot resistance from our field results. Considering that this may reflect the nature of the leaf spot disease complex and the contrasting resistance or susceptibility of a given line to either component disease, we sought to further examine potential leaf spot resistance through controlled bioassays. Early season leaf spot ratings were used as an indicator of lines most likely to be resistant or susceptible to early leaf spot (ELS) before the confounding effect of late leaf spot (LLS) disease onset. Based on these field ratings, eight lines predicted to be ELS susceptible and seven lines predicted to be ELS resistant were inoculated with the pathogen under controlled conditions and their response measured against resistant and susceptible checks.

**Figure 6:**
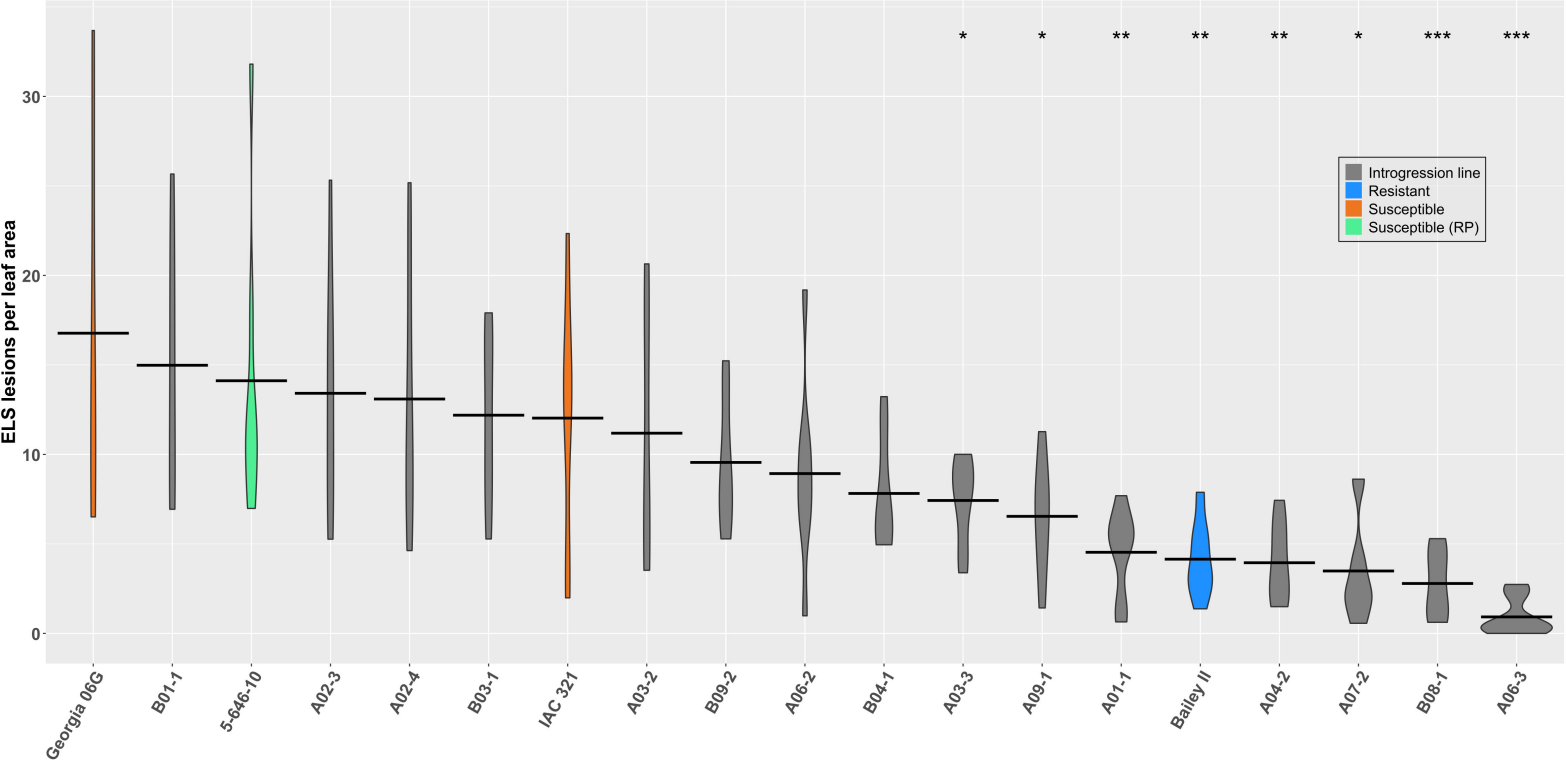
Violin plot of average number of ELS lesions standardized by leaf area for fifteen introgression lines tested (gray bars) against the controls ‘Georgia-06G’ (highly susceptible; orange), ‘IAC 321’ (moderately susceptible, resistant to LLS; orange), ‘Bailey II’ (resistant; blue) and the recurrent parent, ‘5-646-10’ (green). Mean values per genotype are shown by crossbars. Means are compared to the recurrent parent, ‘5-646-10’, by Mann-Whitney U test with significant differences indicated by asterisks above the corresponding violin bar. *p  < 0.05; **p  < 0.01; ***p  < 0.001.

21 days after inoculation, the number of ELS lesions per sample were counted and total leaf area was measured from images collected using a flatbed scanner (see Materials & Methods). Lesion counts were standardized to leaf area to account for varying leaf sizes among genotypes. The recurrent parent line, ‘5-646-10’, performed similarly to the susceptible cultivar ‘Georgia-06G’, as expected, as did the cultivar ‘IAC 321’, which has strong LLS resistance but is moderately to highly susceptible to ELS. The cultivar ‘Bailey II’, released as an ELS-resistant variety and included as a resistant check, showed a mean lesion per leaf area (LPLA) value significantly lower (*p* < 0.01) than ‘5-646-10’. Seven ILs also had a mean LPLA significantly lower than ‘5-646-10’; interestingly, three of these were lines predicted to be susceptible based on field performance, including the line with the lowest mean LPLA, ‘RBS-IL_A06-3’. The four lines that showed reduced lesion development in both bioassay conditions and field conditions were ‘RBS-IL_A03-3’, ‘RBS-IL_A04-2’, ‘RBS-IL_A07-2’, and ‘RBS-IL_B08-1’. Results for all lines are summarized in **Figure 6**.

### 3.5 Drone-based phenotyping of canopy traits correlates with disease ratings

Early-season TSWV ratings (64 DAP) were found to correlate strongly with mean canopy area measurements collected by aerial imagery (*r* = -0.55, *p* = 4.54e-04), with a similarly strong relationship observed at the end of the growing season. Canopy area measurements collected as early as 40 DAP correlate with TSWV ratings made at 64 DAP (*r* = -0.58, *p* = 1.861e-04) and with end-of-season AUDPC values (*r* = -0.48, *p* = 2.81e-4). This may be explained by the stunted phenotype of TSWV-infected plants causing a reduced canopy area. During early-season ratings, the percentage of plants in each plot with no or very minor symptoms was noted, termed the “low incidence” phenotype. This measurement was found to be a strong predictor of end-of-season AUDPC (*r* = -0.60, *p* = 1.28e-4) and to show strong, significant positive associations with NDVI through mid-season (until 96 DAP), when the effects of leaf spot become more prevalent. Because of its potentially useful nature as an early-season indicator of TSWV resistance throughout the season, it is included in the analyses below.

**Figure 7:**
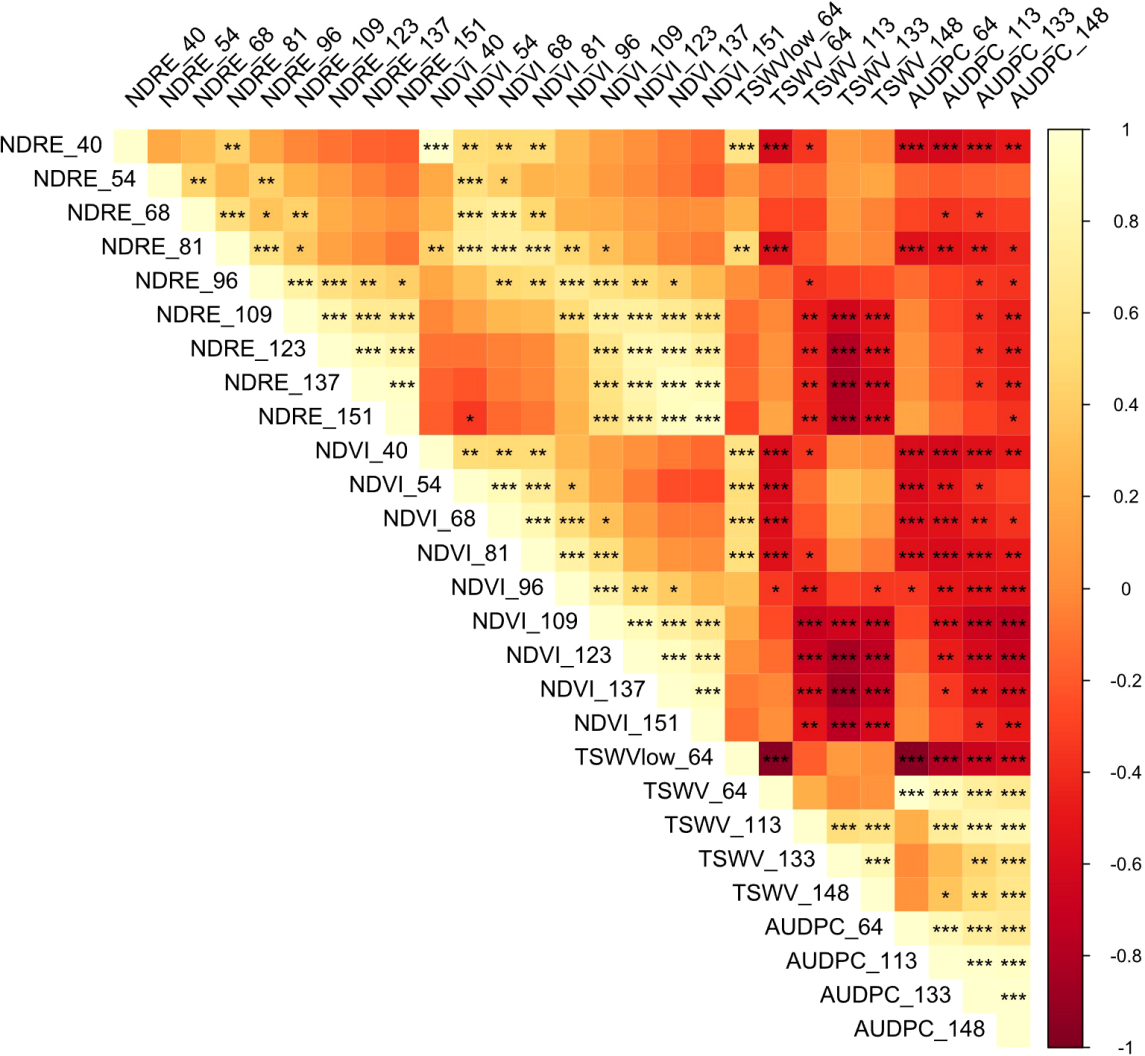
Correlation matrix of measurements of normalized difference of red edge (NDRE), normalized difference vegetation index (NDVI), both collected by UAV, and manually scored tomato spotted wilt virus (TSWV) severity ratings. Numbers indicated in each row/column are days after planting that each phenotype was measured. -1 to 1 color scale represents Pearson correlation coefficient.

Strong and highly significant correlations were additionally found between manual TSWV evaluations and normalized difference vegetation index (NDVI) and normalized difference red edge (NDRE) collected by unmanned aerial vehicle (drone). Between early season evaluations (64 DAP) and late season evaluations (148 DAP), correlation with NDVI values measured within four days of evaluation ranged from -0.56 (64 DAP) to -0.86 (133 DAP). While correlation with NDRE was weak early in the growing season, the relationship was stronger mid-season and especially late-season. Throughout the season, NDVI served as a better predictor for TSWV scores than NDRE index. However, we found both measures to be poor predictors of leaf spot infection. All correlation coefficients between disease rating AUDPC values at each point in the growing season and the three UAV-measured data points discussed here (canopy area, NDVI and NDRE) are presented in **Table 3** and visualized in **Figure 7**.

**Table 3:** Correlations between UAV (drone)-collected phenotypic data and manually scored disease ratings throughout the growing season (DAP, days after planting).

UAV measurement	TSWV, 64 DAP (low incidence)	TSWV, 64 DAP	TSWV, 113 DAP	TSWV, 133 DAP	TSWV, 148 DAP	Leaf spot, 113 DAP	Leaf spot, 133 DAP	Leaf spot, 148 DAP
Canopy area	*r* = 0.59 *p* = 1.55e-4	*r* = -0.55 *p* = 4.54e-4	*r =* -0.52 *p* = 0.00117	*r* = -0.54 *p* = 6.60e-4	*r* = -0.56 *p =* 3.29e-4	*r* = 0.15 *p =* 0.118	*r =* 0.10 *p* = 0.294	*r =* 0.094 *p =* 0.341
NDVI	*r* = 0.54 *p* = 6.19e-4	*r* = -0.56 *p* = 3.99e-4	*r =* -0.70 *p* = 4.91e-6	*r* = -0.86 *p* = 1.37e-11	*r* = -0.60 *p =* 1.05e-4	*r =* 0.0073 *p* = 0.966	*r =* 0.012 *p* = 0.946	*r* = 0.033 *p =* 0.353
NDRE	*r* = 0.23 *p* = 0.168	*r =* -0.28 *p* = 0.096	*r* = -0.47 *p* = 0.00575	*r* = -0.80 *p* = 6.47e-9	*r* = -0.59 *p =* 1.52e-4	*r =* -0.21 *p =* 0.211	*r* = 0.078 *p =* 0.807	*r =* 0.14 *p* = 0.404

Correlation is shown from the flight data closest in time to manual data collection, ± 1–3 days. *R* values presented are Pearson correlation coefficients.

## 4 Discussion

This study describes the successful development of a structured introgression line (IL) population incorporating genomic segments from the wild peanut species *Arachis stenosperma* and *Arachis batizocoi* into a cultivated peanut background. This resource addresses a major challenge of utilizing the genetic diversity of wild relatives in peanut breeding, which is complicated by ploidy barriers, involved interspecific hybridization procedures, and restoring a cultivated-like phenotype. The extensive genome coverage and phenotypic variability observed among the ILs indicate effective introgression of wild alleles with potential value for crop improvement and genetic dissection of complex traits. The utility of this population as a resource for basic trait mapping has been demonstrated through the effective mapping of flower color, seed weight, and pod constriction to genomic locations in agreement with previous findings.

### 4.1 Genomic coverage and introgression dynamics

With this IL population, we have provided coverage of approximately half of the peanut genome with coverage from the wild relatives *A. stenosperma* and *A. batizocoi*. The A subgenome, which recombines with *A. stenosperma*, has approximately twice as much coverage as the B subgenome. While we initially sought to curate a population with 100% coverage, we found that, in field conditions, several lines suffered nearly 100% mortality or yielded so poorly as to be unusable in the next generation. Alternatively, multiple chromosomes were entirely purged of introgressions from the parent population before selection for the IL population began (**Supplementary Figure 1**). Considering the large size and diversity of the parent population, the total loss of introgression from entire chromosomes may similarly suggest a reduction in fitness resulting from introgression on those chromosomes. This effect clearly showed a preferential expulsion of introgressions from *A. batizocoi* in the B subgenome (**Supplementary Figure 2**). This may be explained by *A. batizocoi* introducing alleles that reduce fitness in our growing conditions.

Another potential explanation is weak or inconsistent recombination between *A. batizocoi* and the B subgenome on the molecular level. Cytogenetic analysis has demonstrated that *A. batizocoi* is more distantly related to the progenitor species of the B subgenome than *A. stenosperma* is to the progenitor species of the A subgenome (Leal-Bertioli et al., 2015). Previous reports remark the genetic recombination between *A. batizocoi* and the B subgenome of cultivated peanut (Leal-Bertioli et al., 2018), but do not observe the stability and maintenance of *A. batizocoi* genetics over multiple generations. While *A. batizocoi* recombines with the ‘B’ subgenome of cultivated peanut, it is considered to be a ‘K’ genome species rather than a ‘B’ genome species *sensu stricto* like *A. ipaënsis*, the progenitor of the cultivated B subgenome (Robledo and Seijo, 2010; Leal-Bertioli et al., 2024). *A. batizocoi* and *A. ipaënsis* have distinct karyotypes, and crosses between the two species have been observed to form fewer than the expected 10 bivalents in meiosis that are generally seen in ‘B’ × ‘B’ or ‘K’× ‘K’ crosses (Tallury et al., 2005). GISH has also revealed weak binding between *A. batizocoi* and the B subgenome of cultivated peanut (Seijo et al., 2007). Thus, the divergence between *A. batizocoi* and the B subgenome on the genetic and karyotypic level could be suppressing or destabilizing recombination between some chromosome pairs.

It is important to note that three lines appear to have introgressions from the wild species *Arachis cardenasii*. Line ‘RBS-IL_A04-2’ shows signatures of *A. cardenasii* introgression from SNP genotyping, and ‘RBS-IL_A07-2’ has a characteristic red seed coat seen in *A. cardenasii* and Brazilian breeding lines featuring introgressions from this species such as ‘IAC 321’. These are two of four lines within the population with the breeding line ‘13-2113’ in their parentage; ‘13-2113’ includes *A. cardenasii* genetics, including an RKN resistance locus, and was used as a backcross parent to introduce this source of resistance. In contrast, line ‘RBS-IL_A03-3’ shows a likely introgression from A. cardenasii on chromosome A08, but does not have ‘13-2113’ in its pedigree and may have been cross-pollinated with a hybrid cultivar in field conditions. While these introgressions from *A. cardenasii* may unfortunately complicate or cloud analyses of this IL population, this unintentional hybridization may have introduced additional alleles of interest. The benefits of *A. cardenasii* for peanut improvement have proven to be innumerable and are well-documented (Bertioli et al., 2021a). ‘RBS-IL_A02-3’ and ‘RBS-IL_B09-2’ also have this breeding line in their pedigree but do not appear to have inherited any introgressions from *A. cardenasii*. Genotyping information from *A. cardenasii*-specific markers is given in **Supplementary Table 2**, sheet “*A. cardenasii*”.

### 4.2 Phenotypic diversity and preliminary QTL mapping

The phenotypic diversity observed in the population reflects the genetic diversity that has been introduced from the ‘BatSten1’ donor. As percentages of variation from the average phenotype of the recurrent parent, the IL population phenotypes ranged from -29.3% to 45.0% for plant height; -47.0%, to 2.0% for hundred-seed weight; -24.5%, to 24.5% for pod constriction; -24.2% to 14.2% for percentage double pods; and -58.1% to 36.1% for yield.QTL analysis generally yielded milder PVE values than the total observed variance from the recurrent parent, but results were remarkably similar to those obtained by simple statistical comparison of means of phenotypic values between ILs and the recurrent parent. This attests to the utility of the IL population structure in simplifying complex traits.

Comparison with previous studies utilizing wild peanut relatives provides important context for interpreting the introgression patterns and phenotypic variation observed in the present study. In many cases, QTL that have previously been identified for the traits examined presently are not replicated in this study. Considering most peanut genetic studies of wild introgressions make use of ILs with different wild species introgressed in a genetic background of a different botanical variety (Kassie et al., 2023), this is not entirely surprising, and the validity of previous and present findings are not mutually exclusive. One study previously performed in the same genetic background on pod constriction found a major effect QTL at the top of chromosome B08 (Ballén-Taborda et al., 2024), a result which was replicated in this study. The effect size of this introgression on pod constriction was smaller in the present study (16.9%) than that reported by Ballén-Taborda et al. (2024), who observed an effect size of 47.3%. In that study, the QTL was validated in only two of five families exhibiting strong pod constriction, and the authors suggested that the effect of smaller QTLs may have been confounded with the B08 QTL, consistent with the Beavis effect (Beavis, 1998). Indeed, we did find a smaller-effect QTL on chromosome A03 in this study; however, two of three lines introgressed at this QTL also have the B08 QTL. To better understand the genetic basis of this trait, further study is required.

A 2020 study analyzing pod and seed weight in two nested association mapping populations in peanut found QTLs (chromosome A05, 102.05 – 105.06, 15.25 – 93.32, 97.87 – 97.87 Mbp) for both traits that collocate to *SW_A05_1* (8.44 – 132.86 Mbp) (Gangurde et al., 2020). In this previous study, the highest PVE value for a seed weight QTL was 40.3% while most were in the range of 25-35%; QTLs were also detected on several other chromosomes. Thus, it is likely that the strength of phenotypic effect seen here is inflated by the limited sample size of the study. Interestingly, this region includes a spermidine synthase gene, which was demonstrated in rice to have a strong negative effect on grain size (Tao et al., 2018; Gangurde et al., 2020).

The preliminary QTL mapping done presently is mainly intended as a proof of concept. Small population size and incomplete genomic introgression coverage mean that this tool, as is, is not ideal for QTL mapping experiments which require a high level of sensitivity or fine resolution. This is evinced by the large confidence intervals associated with most QTLs identified in this study. To improve the statistical power of an intended mapping experiment performed with this population, a larger number of replications is recommended. Experimental design that minimizes or accounts for environmental variance will also improve similar experiments. For more refined genetic mapping, the ILs including these QTLs could be used for further backcrossing and the development of a larger recombinant inbred population for a particular trait of interest. Such an approach has been used for a pod/seed size reduction QTL from the [*A. ipaënsis* × *A. duranensis*]^4x^ CSSL population, allowing the QTL to be narrowed to a 168 kb chromosomal region of 22 genes (Alyr et al., 2020).

### 4.3 Leaf spot, TSWV resistance and high-throughput phenotyping

**Figure 8:**
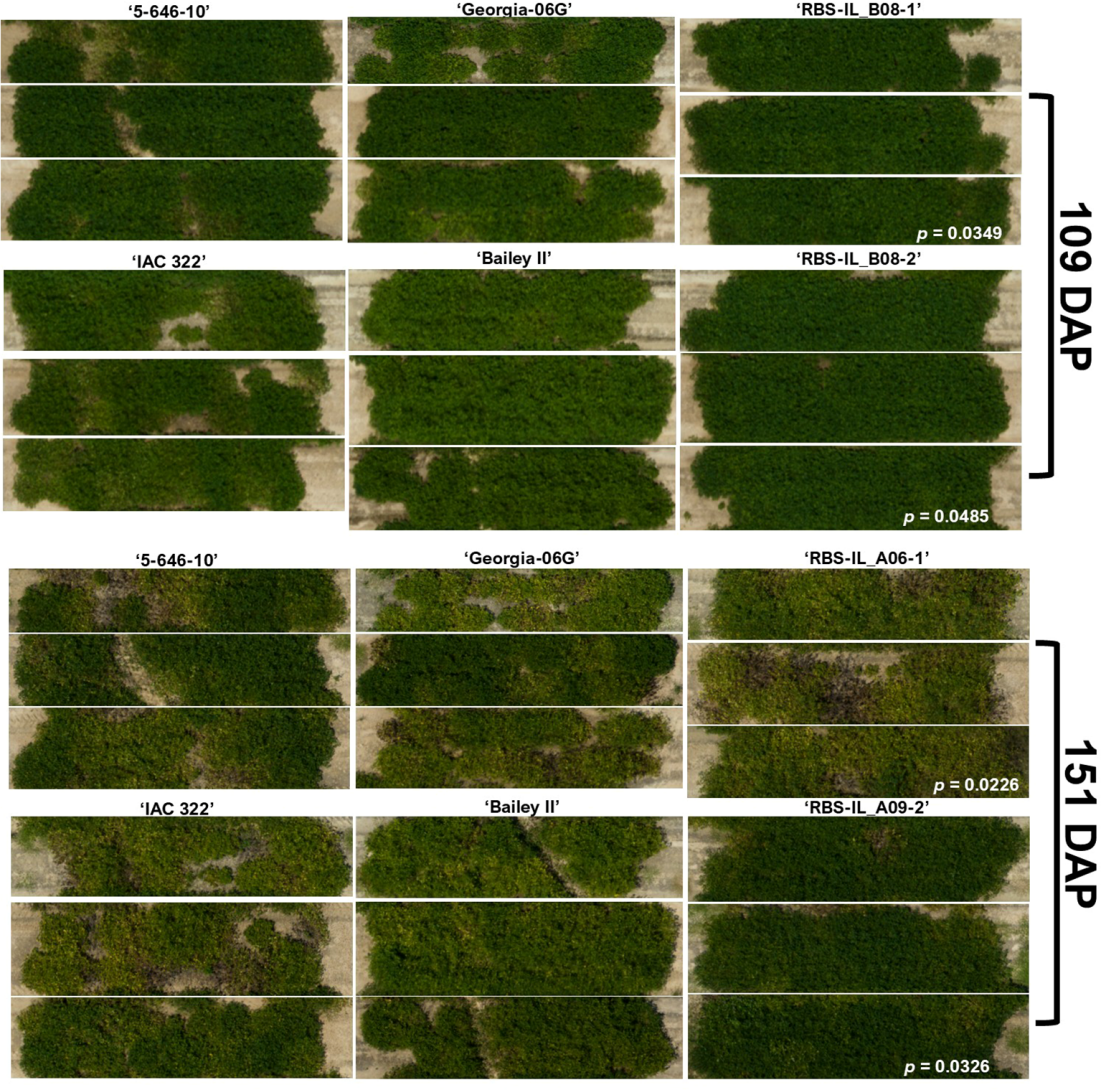
Aerial imagery reveals vigor (‘RBS-IL_B08-1’, ‘RBS-IL_B08-2’, and ‘RBS-IL_A09-2’) and heightened susceptibility (‘RBS-IL_A06-1’) among introgression lines against mid-season (predominantly TSWV and ELS) and late-season (TSWV and LLS) disease pressure compared to four cultivated controls. ‘5-646-10’ is the recurrent cultivated parent of the IL population; ‘Georgia-06G’ is the most popular cultivar in the Southeast growing region; ‘IAC 322’ is the sister line to a Brazilian cultivar released for LLS resistance (but ELS and TSWV susceptible); ‘Bailey II’ is an ELS-resistant Virginia-type cultivar. *P*-values indicated are pairwise comparisons of NDRE (normalized difference red edge index, a measure of healthy vegetation) by *t*-test to the recurrent parent, ‘5-646-10’.

The population shows promising results for two major diseases of peanut production in the United States, TSWV and ELS. While the scale used for TSWV rating (0–5 with increments of 0.5) combined with a relatively high level of variability across a low number of field replicates limited the statistical power of this metric, both lines with introgressions on chromosome B08, including a small overlapping region, performed noticeably better against TSWV (average end-of-season scores of 2.17 ± 0.17 and 2.50 ± 0.29, respectively) than did the recurrent parent, ‘5-646-10’ (average score 3.33 ± 0.44) or leading cultivar ‘Georgia-06G’ (average score 3.50 ± 0.29). These lines also outperformed ‘Bailey II’ (average score 3.17 ± 0.17). This is notable considering ‘Georgia-06G’ and ‘Bailey II’ are both considered to be TSWV-resistant cultivars (Branch, 2007; Isleib, 2011). While field conditions can make it challenging to isolate the influences of diseases with similar symptomology and overlapping times of prevalence, the relative vigor of some of these introgression lines can be appreciated visually in **Figure 8**.

The correlation noted between manual TSWV ratings and drone-collected measures of canopy area and NDVI/NDRE indices may be a useful point of investigation for future efforts to develop high-throughput disease resistance screening methods. We found the stronger correlation with manual ratings to be obtained from NDVI values than NDRE, in contrast to previous findings (Patrick et al., 2017); however, both were strongly negatively correlated and highly significant (**Table 3**). The magnitude of the correlation between NDVI values and manual TSWV ratings made at 133 DAP seen in this study, *r* = -0.86, was very similar to that reported by Patrick et al. (2017) at 120 DAP (-0.90) and Gimode et al. (2023). at 110 DAP (-0.86). In agreement with previous findings (Patrick et al., 2017), our data show that early-season phenotyping correlates with end-season disease rating AUDPC (*r* = -0.37, *p* = 0.0281), although the strength of the correlation and predictive value increases greatly around 96 DAP (*r* = -0.53, *p* = 8.268e-4) (**Figure 7**). In contrast to Gimode et al.’s (2023) findings, there was no correlation between drone-collected vegetative indices and leaf spot ratings in this study (**Table 3**).

Four lines were found to demonstrate moderate to strong ELS resistance in controlled bioassay conditions and early-season field performance consistent with ELS resistance. Interestingly, one of these was ‘RBS-IL_B08-1’, which was among the most strongly resistant lines in field conditions to both early season LS and TSWV as well as the ELS bioassay. This line showed more severe late season LS symptoms, however, and may likely be susceptible to LLS. Another line identified as resistant in both the ELS bioassay and early season LS ratings was ‘RBS-IL_A03-3’. Previous publications have also reported ELS resistance mapped to chromosome A03, introgressed from *A. cardenasii* (Han et al., 2018; Chu et al., 2019). Chu et al. (2019) found that the distal region (140.08 – 142.61 Mbp, physical positions updated according to most recent SNP array and cultivated reference genome (Bertioli et al., 2019; Korani et al., 2019)) of chromosome A03 conferred resistance to both ELS and LLS, neighboring the region covered by ‘RBS-IL_A03-3’ (144.67 – 144.82 Mbp). While introgressions from a different species in a different population cannot be expected to map to the same genetic position, the relatively high sequence identity between *A. cardenasii* and *A. stenosperma* and the frequent clustering of disease resistance R genes in common genomic regions makes it a reasonable expectation that these similar findings may indicate homologous resistance loci. Similarly, ELS resistance loci from *A. cardenasii* were identified in a central region of chromosome A07 (Han et al., 2018), similar to ELS-resistant ‘RBS-IL_A07-2’, although direct comparison is difficult without physical positions or marker names available to compare genetic maps between the two studies.

### 4.4 Application in peanut breeding

Crop wild relatives (CWRs) are a fundamental resource for crop improvement. While their utility in plant breeding has been recognized for several decades (Hodgkin and Hajjar, 2007; Prescott-Allen, 1986), it is only since the 1980s that efforts to prioritize the description, preservation and utilization of CWRs have gained considerable momentum (Dempewolf et al., 2017). Two major reasons for their relatively delayed and underwhelming adoption despite their widely acknowledged high level of genetic value is that CWRs often have limited sexual compatibility with their related cultivated species, and successfully recovered progeny often suffer some extent of linkage drag between the desired wild trait and reduced agronomic performance and/or loss of the cultivated phenotype (Bohra et al., 2022).

Introgression lines (ILs) largely or completely address both concerns by providing small amounts of CWR-derived alleles in a genetic background compatible with cultivated species. Moreover, structured populations of ILs provide maximal genome coverage across the cultivated species with wild-derived introgressions, discretized across several lines, each of which are highly compatible with the cultivated species. In addition to far greater ease of use compared to the initial CWR materials, this structure has the benefit of “Mendelizing” the effect each introgression has even on complex traits. This can be appreciated in the strong results seen for polygenic traits such as hundred-seed weight and plant height in field evaluations of the present population. With this population structure, QTL analysis can be performed without genotyping, providing a valuable tool for resource-limited breeding programs.

Among the phenotypes seen in the population which may find immediate application in peanut breeding is the increased plant height more commonly typical of Virginia-type cultivars seen in line ‘RBS-IL_B01-2’ (avg. 43.1 ± 2.0 cm; ‘Bailey II’ 47.8 ± 1.7 cm, recurrent parent ‘5-646-10’ 29.7 ± 1.1 cm) or, perhaps more pertinently, the increased seed size of line ‘RBS-IL_A06-1’ (avg. 72.4 ± 0.45 g; ‘Bailey II’ 81.3 ± 4.70 g, recurrent parent ‘5-646-10’ 68.5 ± 0.81 g). While these lines currently represent something of an intermediate phenotype between typical runner- and Virginia-type peanuts, they may be useful resources in meeting market interest in a large-seeded Virginia-style cultivar for the Southeast region, where climactic conditions favor runner-type peanuts.

In addition to TSWV and ELS, previous studies conducted in the diploid species would suggest that they have introduced in the population resistance against diseases that were not assayed in the present study (e.g., Subrahmanyam et al., 2001; Arias et al., 2018; see also Seijo et al., 2007). It is also possible that additional resistance alleles to the pathogens discussed are present in the population that evaded detection by the relatively small disease trials conducted here. It is our hope that peanut pathologists and breeders targeting in genetic disease resistance will use these materials to perform more thorough examinations of potential resistance to their pathogen of interest. Another potential avenue of research not investigated here, but in which peanut wild relatives have been identified as particularly useful, is adaptation to abiotic stressors such as heat stress and drought, which are increasingly sought-after crop protection traits (Dutra et al., 2018; Seijo et al., 2007; Leal-Bertioli et al., 2012; Guimarães et al., 2012; Dempewolf et al., 2017; Bohra et al., 2022). Examining this IL population for these and other climate resilience traits is recommended. We believe that, in a similar fashion to the application of *A. cardenasii* genetics to addressing local issues of peanut cultivation across several continents (Bertioli et al., 2021a), this population is likely to find breeding applications in far-reaching and perhaps unexpected places if it is adopted by the community.

### 4.5 Limitations and future directions

The development of this population was hampered by the failure to recover introgression lines with coverage of chromosomes A08, B02, B05, B06 and B10. In selecting lines to cover the remaining chromosomes, a decision was made to favor a smaller number of lines, so that it is easy to phenotype the full population, and lines with limited ‘off-target’ introgressions to preserve the “Mendelizing” effect of the population structure as much as possible. As a consequence, some chromosomes that are represented in the population are only partially covered by introgressions and many introgressions are dozens of megabases in size. While the more compact set of 32 lines has certain advantages, it may be advantageous in the future to supplement this “core” population with supplemental ILs that maximize genome coverage in the currently absent regions, even if greater levels of background introgression are present. Even more, backcross efforts could be begun again from ‘BatSten1’ to recover these “missing” ILs. Without the development of additional lines to be added, the population could be improved by the development of additional SNP markers to better characterize the introgressions present in the population. Chromosomes A10 (117.08 Mbp) and B10 (145.03 Mbp) are only represented by 13 and 11 polymorphic SNPs, respectively, for example; thus, introgressions on these chromosomes or in other marker-sparse regions may go undetected or underrepresented.

Regarding the disease resistance trials and agronomic trait QTL mapping performed in this study, results are limited in their interpretation due to the inherent variability and low number of replications (*n* = 3) imposed as a practical constraint on our field trials. In many cases, the confidence interval spans the entire introgression in which the QTL lies or even most of the chromosome, particularly on marker-sparse chromosomes; improved marker density would also strengthen mapping experiments. To reliably characterize the putative disease resistance loci identified presently will require more thorough study, ideally through both multilocation field trials and more extensively replicated bioassay experiments. It will also be worthwhile to assay the full population for resistance to ELS, as only a subset of lines was examined here.

## 5 Conclusion

Here we report the construction of a new IL population for peanut that introduces the genetics of two wild relatives, *Arachis stenosperma* and *Arachis batizocoi*. The more distant relationship between the donor species and the subgenomes of cultivated peanut may underlie the loss of ‘B’ subgenome introgressions, but diversifies the primary gene pool. We have shown the utility of the population in identifying genetic regions underlying qualitative and quantitative traits. Our findings are generally compatible with reports of QTL for these traits in other populations. Several potentially useful loci have been identified, particularly for plant height and disease resistance. Moreover, this IL population provides a permanent resource for peanut breeders that allows the rapid deployment of alleles from the two donor species into their breeding program. The nature and number of the alleles introduced is far from being quantified presently; indeed, the greatest value of this introgression line population may lie in providing solutions to problems that are yet unknown to the peanut breeding and research community. By submitting this population for public dissemination, we encourage the broader community to exploit this resource for any application where it may be of use.

## Data Availability

The datasets presented in this study can be found in online repositories. The names of the repository/repositories and accession number(s) can be found in the article/**Supplementary Material**.

## Supplementary material

Supplementary Figure 1 Mean introgression coverage across the genome through progressive generations in the parent ‘BatSten1’ population. Chromosomes B05, B06 and B10 (as well as A08) were fully eliminated by the BC2F2 generation as well as almost all of chromosomes B02, B07 and B08. Since genome-wide negative background selection was not begun until the BC2F2 generation, this suggests a fitness disadvantage or cytogenetic reason for the purging of B subgenome introgressions.

Supplementary Figure 2 Relative depletion and enrichment of introgression coverage in homeologous chromosomes in subsequent generations of the ‘BatSten1’ parent population. In each panel, mean introgression coverage is shown for each chromosome in the **(A)** (left) and **(B)** (right) subgenomes. Negative-sloping lines represent a relative depletion in the B subgenome, whereas positive-sloping lines represent B chromosomes with greater introgression coverage. The size of the dots of the B subgenome are scaled to the change in coverage relative to the homeologous A chromosome. Dotted lines show mean introgression coverage expected according to Mendelian inheritance assuming no selection in each generation. * p < 0.05; ** p < 0.01; *** p < 0.001 according to Wilcoxon rank-sum test.
