# Supplementary figures and images for: Construction of an introgression line population for cultivated peanut (Arachis hypogaea) to facilitate breeding with wild relatives Arachis batizocoi and Arachis stenosperma
Source: Front Plant Sci. 2026 May 1;17:1799510. doi: 10.3389/fpls.2026.1799510 (PMC13176233; doi:10.3389/fpls.2026.1799510)

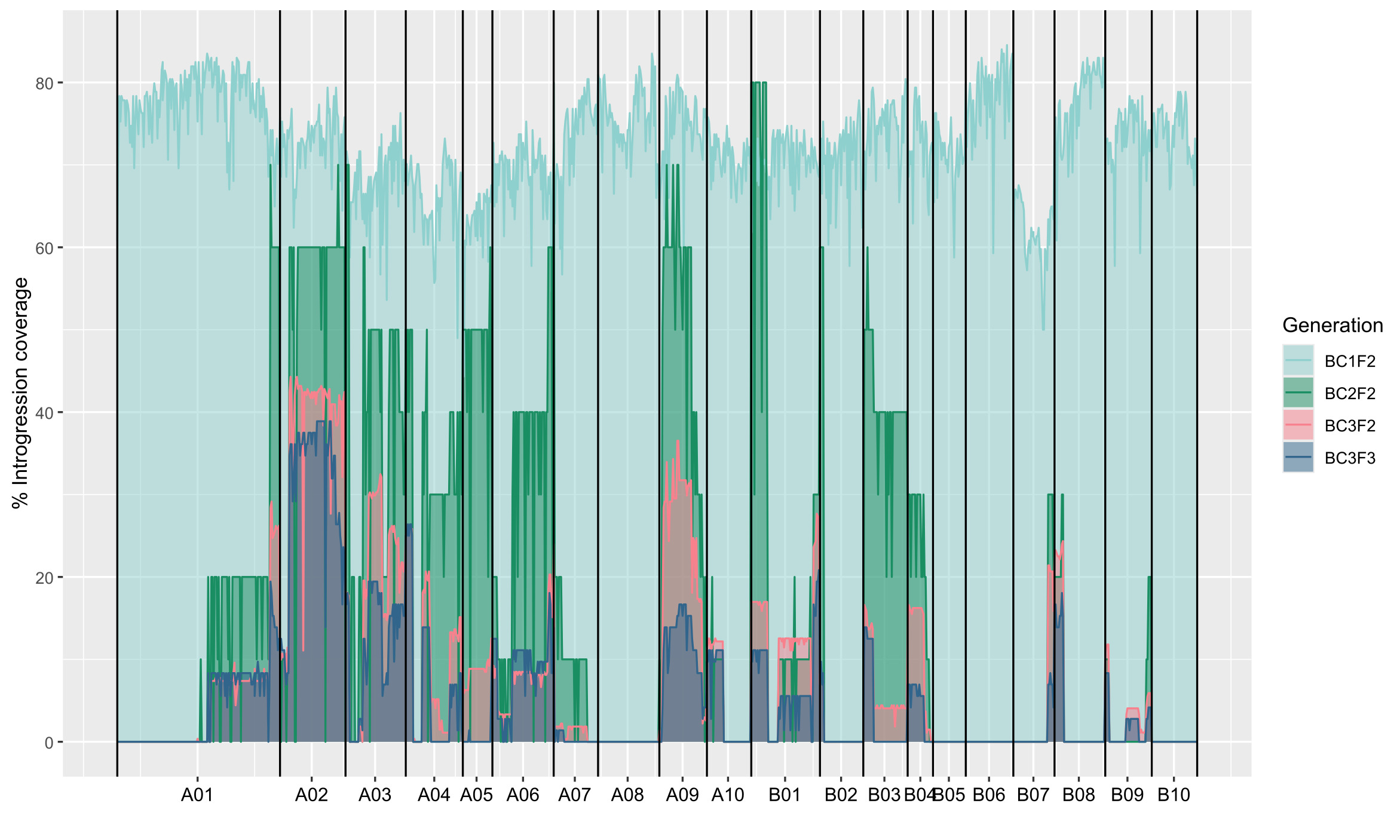

Supplement: Supplementary Figure 1 — Mean introgression coverage across the genome through progressive generations in the parent ‘BatSten1’ population. Chromosomes B05, B06 and B10 (as well as A08) were fully eliminated by the BC2F2 generation as well as almost all of chromosomes B02, B07 and B08. Since genome-wide negative background selection was not begun until the BC2F2 generation, this suggests a fitness disadvantage or cytogenetic reason for the purging of B subgenome introgressions. [file Image1.jpeg]

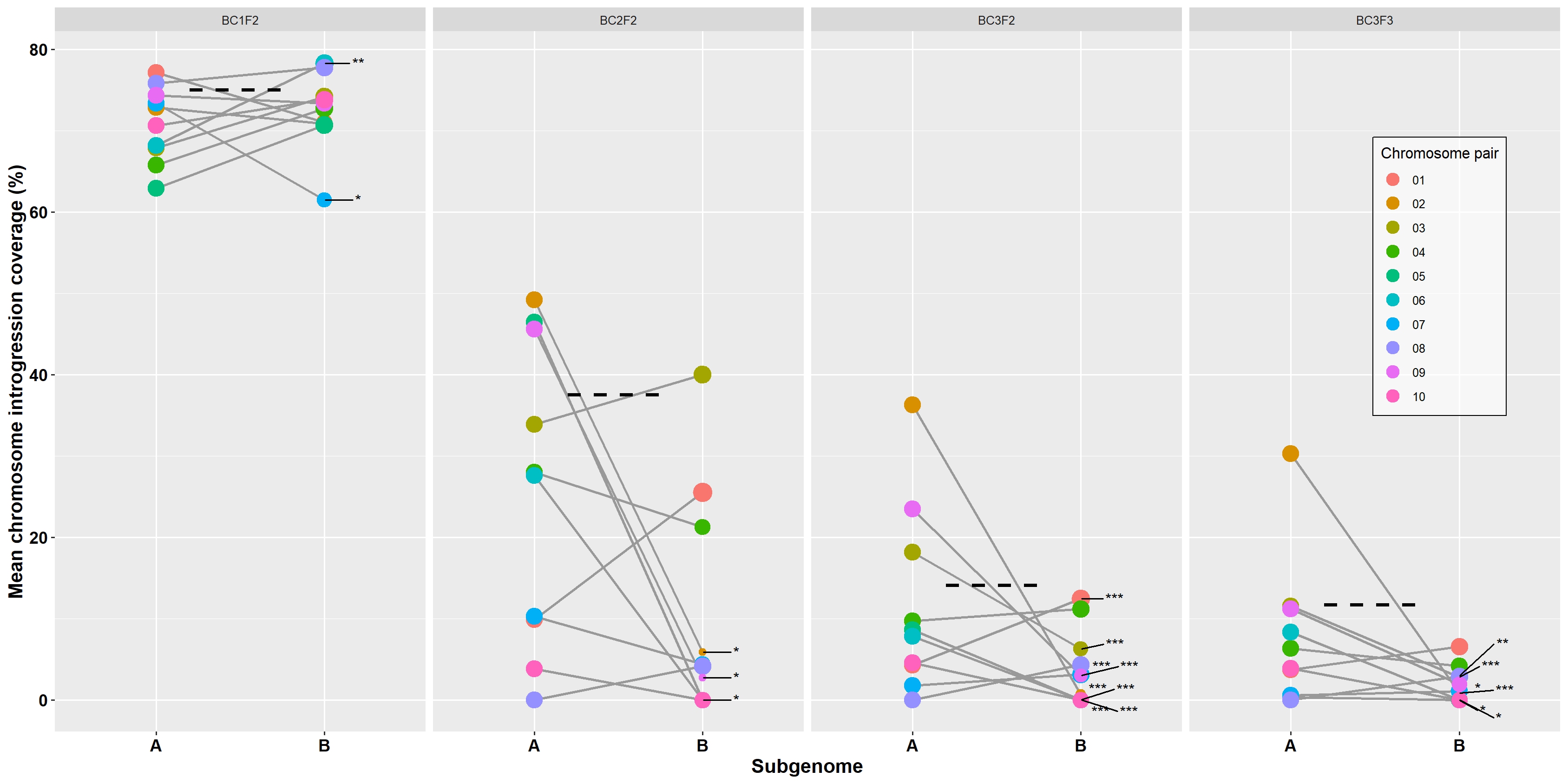

Supplement: Supplementary Figure 2 — Relative depletion and enrichment of introgression coverage in homeologous chromosomes in subsequent generations of the ‘BatSten1’ parent population. In each panel, mean introgression coverage is shown for each chromosome in the (A) (left) and (B) (right) subgenomes. Negative-sloping lines represent a relative depletion in the B subgenome, whereas positive-sloping lines represent B chromosomes with greater introgression coverage. The size of the dots of the B subgenome are scaled to the change in coverage relative to the homeologous A chromosome. Dotted lines show mean introgression coverage expected according to Mendelian inheritance assuming no selection in each generation. * p < 0.05; ** p < 0.01; *** p < 0.001 according to Wilcoxon rank-sum test. [file Image2.jpeg]
